# Optimum Inhibition of Amphotericin-B-Resistant *Candida albicans* Strain in Single- and Mixed-Species Biofilms by *Candida* and Non-*Candida* Terpenoids

**DOI:** 10.3390/biom10020342

**Published:** 2020-02-21

**Authors:** Hidaya F. Z. Touil, Kebir Boucherit, Zahia Boucherit-Otmani, Ghalia Kohder, Mohamed Madkour, Sameh S. M. Soliman

**Affiliations:** 1 Laboratory Antibiotics Antifungals: Physico-Chemical, Synthesis and Biological Activity (LapSab), Tlemcen University, Tlemcen B.P 119, Algeria; hidayet.touil@gmail.com (H.F.Z.T.); boucheritkebir@yahoo.fr (K.B.); z_boucherit@yahoo.fr (Z.B.-O.); 2 University Center Belhadj Bouchaïb, Aïn Temouchent BP 284, Algeria; 3 Sharjah Institute for Medical Research, University of Sharjah, Sharjah PO. Box 27272, UAE; gkhoder@sharjah.ac.ae (G.K.); mmadkour@sharjah.ac.ae (M.M.); 4 Department of Medicinal Chemistry, College of Pharmacy, University of Sharjah, Sharjah PO. Box 27272, UAE; 5 Department of Medical Laboratory Sciences, Collage of Health Sciences, University of Sharjah, Sharjah PO. Box 27272, UAE; 6 Department of Pharmacognosy, College of Pharmacy, University of Zagazig, Zagazig 44519, Egypt

**Keywords:** highly resistant *Candida albicans*, mixed infection, biofilm, terpenoids, farnesol, carvacrol

## Abstract

*Candida albicans* is one of the most common human fungal pathogens and represents the most important cause of opportunistic mycoses worldwide. Surgical devices including catheters are easily contaminated with *C. albicans* via its formation of drug-resistant biofilms. In this study, amphotericin-B-resistant *C. albicans* strains were isolated from surgical devices at an intensive care center. The objective of this study was to develop optimized effective inhibitory treatment of resistant *C. albicans* by terpenoids, known to be produced naturally as protective signals. Endogenously produced farnesol by *C. albicans* yeast and plant terpenoids, carvacrol, and cuminaldehyde were tested separately or in combination on amphotericin-B-resistant *C. albicans* in either single- or mixed-infections. The results showed that farnesol did not inhibit hyphae formation when associated with bacteria. Carvacrol and cuminaldehyde showed variable inhibitory effects on *C. albicans* yeast compared to hyphae formation. A combination of farnesol with carvacrol showed synergistic inhibitory activities not only on *C. albicans* yeast and hyphae, but also on biofilms formed from single- and mixed-species and at reduced doses. The combined terpenoids also showed biofilm-penetration capability. The aforementioned terpenoid combination will not only be useful in the treatment of different resistant *Candida* forms, but also in the safe prevention of biofilm formation.

## 1. Introduction

*Candida albicans* is the fourth most common cause of nosocomial bloodstream infection [1,2]. *C. albicans* infections are common among hospital patients and elderly people, and are difficult to control [3]. About 50% of adult people have *C. albicans* yeasts in their mouths, and it is responsible for superficial, easily treated infections. However, *C. albicans* infections can spread throughout the body and become life-threatening, particularly with immune-compromised patients [4]. Candidiasis represents a major cause of morbidity and mortality in a broad range of immune-compromised patients [5]. *C. albicans* infections are very difficult to treat due to their resistance to antifungal drugs, expression of virulence factors, and ability to form biofilms. *C. albicans* can switch between two forms, the yeast and hyphae forms. The switch from yeast to hyphae is a recognized virulence factor of *C. albicans* [6], since it is mainly associated with drug-resistant biofilm formation. Most candidiases are associated with the formation of biofilms at a wide range of implanted medical devices [7].

Biofilm-related infections caused by *C. albicans* represent a major threat to public health, as these infections are inherently resistant to most antifungal treatments and thus constitute a reservoir for continued infection [8]. Biofilms are usually found on medical devices such as prostheses, cardioverter defibrillators, urinary and vascular catheters, and cardiac devices [9].

*C. albicans* biofilm exists as complex, diverse, and heterogeneous structures [10] that may be complicated by bacterial infections. These poly-microbial structures (*C. albicans*/bacteria) can ultimately influence disease severity by promoting intensified pathogenic phenotypes [11], including an increased in resistance to both host defenses and antimicrobial therapies. There are increasingly few antimicrobials available to treat such life-threatening infections [12]. Thus, screening for new antimicrobials is necessary.

**Figure 1:**
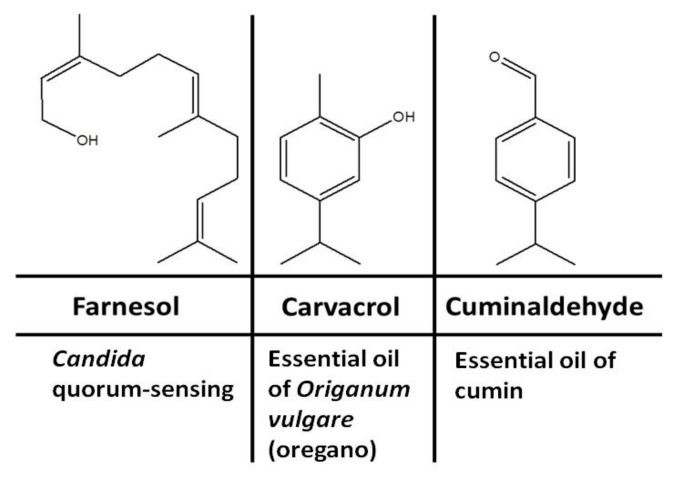
Structures and possible sources of terpenoidal compounds.

Medicinal plants, in particular our daily used plants and condiments, are known sources of antimicrobial terpenoids. Terpenoids are signaling metabolites produced naturally for the purpose of protection. Several terpenoids have been proven to have significant antimicrobial properties [13,14]. Several terpenoids have been shown to exhibit antimicrobial activities by inhibiting morphogenesis, adhesion, and biofilm formation of *C. albicans* [15], biofilm formation and elastase production by *Pseudomonas aeruginosa* and *Staphylococcus aureus* [16], and production of native quorum-sensing compounds [17]. Studies of different terpenoids have revealed that several terpenoid candidates show significant antifungal activity [15], including inhibition of the yeast-to-hyphal transition and biofilm formation in *C. albicans*. The aim of this study was to develop an optimal inhibition strategy for amphotericin-B-resistant *C. albicans* strains using natural volatile terpenoids. In this study, we tested the anti-*Candida* inhibitory effects of three terpenoidal compounds including farnesol, carvacrol, and cuminaldehyde (Figure 1). Farnesol is produced endogenously by *C. albicans* yeast and exogenously in plant foods such as corn cobs. Carvacrol and cuminaldehyde are produced in plant foods and condiments such as oregano and cumin, respectively.

## 2. Materials and Methods

### 2.1. Bacteria and Candida Strains

Fifteen clinical *C. albicans* and 15 clinical bacterial isolates, as listed in Table 1, isolated from medical devices at intensive care department of Tlemcen University Hospital and identified according to Touil et al. (2018), were employed [18]. *C. albicans* strain ATCC10231 was employed as a reference strain.

### 2.2. Determination of the Minimum Inhibitory Concentration (MIC)

The antimicrobial susceptibility of *C. albicans* and bacterial isolates to terpenoidal compounds was determined using the broth microdilution method according to the Clinical and Laboratory Standards Institute (CLSI) for yeast [19] and bacteria [20]. Terpenoidal compounds including farnesol, carvacrol, and cuminaldehyde (Figure 1) and antimicrobial agents including amphotericin B, colistin, and vancomycin were purchased from Sigma Company.

*C. albicans* biofilm was developed according to well-established protocols [21]. Briefly, 200 µL RPMI-1640 containing 5 × 10^5^ cells/mL was incubated in polystyrene, round-bottomed, 96 well microtiter plates (Corning Inc., Corning, NY, USA) (Costar^®^). Terpenoids were applied in the concentrations 1, 2, 3, and 4 mg/mL for cuminaldehyde, 0.25, 0.5, 1, 2 and 3 mg/mL for carvacrol, and 0.22 mg/mL to 66.7 mg/mL for farnesol [22]. The plates were then incubated at 37 °C for 4, 24, and 48 h time periods. The microbial growth was measured at OD_570_ using a microplate reader (BioTekEL × 808, Agilent, Winooski, VT, USA). Clear wells with the lowest terpenoid concentrations and with no visible growth were considered to represent the minimum inhibitory concentration (MIC).

Amphotericin B was employed as a positive control against *C. albicans*. Cultures without terpenoids or antimicrobial agents were employed as negative controls.

### 2.3. Determination of the Minimum Fungicidal/Bactericidal Concentrations (MFC/MBC)

The two wells containing compound concentrations above the MIC were used to determine the minimum fungicidal concentration (MFC) and minimum bactericidal concentration (MBC). A measure of 20 µL from each well was spread on Sabouraud dextrose agar and LB agar and incubated at 37 °C for 24 h. The MFC and MBC were defined as the lowest concentrations that showed no growth [23].

### 2.4. Antimicrobial Activity of Terpenoidal Compounds Combinations against C. albicans and Bacteria

The efficacy of combinations of terpenoidal compounds including cuminaladehyde/carvacrol and farnesol/carvacrol was analyzed in terms of fractional inhibitory concentration indices (FICI) using a checkerboard assay. A measure of 50 µL of each compound was dispensed into a 96 well microtiter plate. A 100 µL yeast and/or bacterial inoculum was added to obtain a final inoculum concentration of 5.0 × 10^5^ CFU/mL. The plates were incubated at 37 °C for 24 h, followed by reading at OD_570_. The concentrations of combined compounds used were selected based on their MIC value (~ 1/2 MIC, 1/4 MIC, 1/8 MIC, and 1/16 MIC). Terpenoid interactions were classified as synergistic, indifferent, or antagonistic on the basis of FICI [24].

### 2.5. Inhibition of Single-Species and Mixed-Species Biofilm Formation

Single- and mixed-species biofilms of *C. albicans* and bacteria were formed in 96 well microtiter plates (Costar^®^, Taufkirchen, Munich, Germany). An aliquot of 100 μl of yeast cell suspension was added in the case of single-species biofilms, or 50 μl of each yeast and bacterial cell suspension for mixed-species biofilms. The inocula were adjusted in RPMI-1640 to a concentration of 1 to 5 × 10^6^ yeast/mL and 1 to 5 × 10^8^ bacteria/mL [25]. The plates were incubated for 4 h at 37 °C. The supernatants were removed and the biofilms formed were washed twice with PBS (pH 7.2) to remove non-adherent cells. RPMI medium (100 µL) containing the terpenoidal compounds was added over the adhered cells and the plates were further incubated at 37 °C for 24 h. The influence of terpenoids on mature biofilms was assessed as follows: 24 or 48 h biofilms were treated with serial concentrations of the terpenoidal compounds (~MIC, 1.5 MIC, and 2 × MIC) and the plates were further incubated at 37 °C for another 24 h. The supernatant including terpenoidal compounds was removed and the fungal viability was analyzed via 3-(4,5-dimethylthiazol-2-yl)-2,5- diphenyl tetrazolium bromide (MTT) assay [14]. The MIC of the terpenoidal compounds that caused 50% and 80% inhibition of *C. albicans* biofilm formation was determined by measuring the metabolic activity of biofilm compared to positive and negative controls.

### 2.6. MTT Assay

The metabolic activity of *C. albicans* cells was evaluated using 3-(4, 5-dimethylthiazolyl-2)-2, 5-diphenyltetrazolium bromide (MTT) assay [26,27] as follows: 96 well microtiter plates were seeded with *Candida* cells. Terpenoids were applied on the seeded cells. Amphotricin B and DMSO were employed as positive and negative controls, respectively. After treatment, the plates were incubated for 24 or 48 h prior to analysis using MTT cell proliferation assay. A measure of 20 µL MTT reagent was added to each well. The plates were incubated for 24 h and the liquid was then discarded, followed by the addition of 200 µL DMSO. The developed purple color was then measured using a Multiskan Go machine spectrophotometer (Thermo Fisher Scientific, Ratastie, Finland) at 570 nm. A higher absorbance and color intensity indicated a higher activity and viability of the cells. Each experiment was repeated six times.

### 2.7. Effect of Terpenoidal Compounds on C. albicans Cell Morphology Using Light Microscopy

In order to assess the compounds’ effects on the architecture of single- and mixed-species biofilms formed by *C. albicans* and bacteria, biofilms produced by three representative *C. albicans* strains (ATCC10231, A5, and A6) were visualized under an inverted microscope (Olympus Life Sciences, Amsinckstraße, Hamburg) at 400 × magnification, following 4 h, 8 h, 16 h, and 24 h exposure to terpenoidal compounds.

### 2.8. Statistical Analysis

The data were graphed using Graph Pad 5.0 for Windows (GraphPad Software, La Jolla, CA, USA). The statistical significance was analyzed by one-way analysis of variance (ANOVA) using either Bonferroni’s multiple comparisons test or Dunn′s multiple comparison test. A *p-*value < 0.05 was considered significant.

### 2.9. Ethics Statement

The authors confirm that the adherence to the ethical policies of the journal. Ethical approval and appropriate consent form were obtained as per the Tlemcen University Hospital rules.

## 3. Results

### 3.1. Amphotricin-B-Resistant C. albicans Clinical Isolates

Our recent data indicated that the MIC of amphotericin B for the planktonic forms of *C. albicans* clinical isolates ranges between 0.25–1 µg/mL, and the biofilm formed from all tested strains showed resistance to amphotericin B [18]. High concentrations up to 32 and 64 µg/mL of amphotericin B were required to inhibit the single-species biofilms formed by *C. albicans* A5 and A6 strains [18], compared to 4 µg/mL required to inhibit the biofilm formed by ATCC10231 strain. On the other hand, we found that the mixed-species biofilm was highly resistant, since > 32 and 64 µg/mL amphotericin B failed to inhibit the biofilms formed by *C. albicans* A5 and A6 strains when mixed with two co-isolated bacteria; *Providencia stuartii* 3 and *Staphylococcus aureus* 6. The SMIC_50_ values of amphotericin B for *C. albicans* A5 and A6 strains in mixed species were 45 and 78 µg/mL.

The aim of this study was to develop an optimal inhibition strategy using natural volatile terpenoids for amphotericin-B-resistant *C. albicans* A5 and A6 strains in either single- or mixed-species biofilms.

### 3.2. C. albicans Inhibition by Endogenous Terpenoid (Farnesol) Was Hyphae-Phenotype-Specific

**Figure 2:**
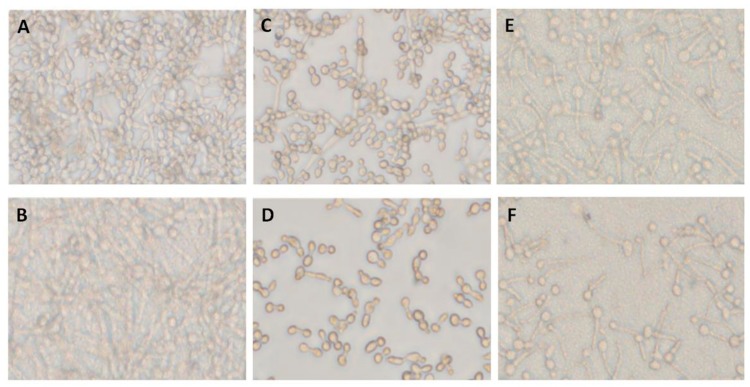
Inhibitory effect of farnesol on single- and mixed-species *C. albicans* biofilm formation. (**A**) *C. albicans* biofilm formed after 12 h (single-species biofilm A5) (untreated biofilm). (**B**) *Candida* biofilm generated by mixed species including *C. albicans* A5, *P. stuartii* 3, and *S. aureus* 6 (A5/Ps3/Sa6). (**C**) Single-species biofilm formed by A5 treated with farnesol at 0.7 mg/mL. (**D**) Single-species biofilm formed by A5 treated with farnesol at 1.4 mg/mL. (**E**) The effect of farnesol at 0.7 mg/mL on biofilm formed by mixed species including *C. albicans* A5, *P. stuartii* 3, and *S. aureus* 6 (A5/Ps3/Sa6). (**F**) The effect of farnesol at 1.4 mg/mL on biofilm mixed-species biofilm formed by *C. albicans* A5, *P. stuartii* 3, and *S. aureus* 6 (A5/Ps3/Sa6).

Pre-adhered *C. albicans* A5, either alone (Figure 2A) or mixed with two co-isolated bacteria, *Providencia stuartii* 3 and *S. aureus* 6 (Figure 2B), were treated with farnesol at 0, 0.7, and 1.4 mg/mL for 12 h. Farnesol showed selective inhibition of hyphae formed by *C. albicans* A5 incubated alone (Figure 2C,D), but not when mixed with co-isolated bacteria (Figure 2E,F), suggesting a decreased susceptibility to farnesol in mixed-species biofilms. Although a concentration of 1.4 mg/mL farnesol could inhibit *Candida* hyphae formation, 12–50 times that concentration was required to stop *C. albicans* yeast growth (Supplementary Table S1), suggesting a selective activity against *C. albicans* hyphae formation.

### 3.3. C. albicans Inhibition by Plant Terpenoids

The effect of two different plant terpenoids, carvacrol and cuminaldehyde (Figure 1), was tested on *C. albicans* yeast and the MICs were determined (Supplementary Table S2). The MIC and MFC of carvacrol were determined to be 0.25–1 and 0.5–2 mg/mL, respectively, and those of cuminaldehyde were 2–4 mg/mL depending on the *Candida* isolates (Supplementary Table S2 and Supplementary Figure S1). *C. albicans* isolates showed higher susceptibility to carvacrol than cuminaldehyde. Carvacrol and cuminaldehyde also showed inhibitory effects on bacteria co-isolated with *C. albicans* at MIC values of 1 mg/mL and 1–4 mg/mL, respectively (Supplementary Table S3).

The effect of combining carvacrol and cuminaldeyde was also tested on *C. albicans* yeast and co-isolated bacteria (Supplementary Table S4). Carvacrol and cuminaldehyde were combined in concentrations lower than their individual MIC values, as indicated in Supplementary Tables S2 and S3. The growth inhibition was determined and FICs were calculated. The FIC index indicates the nature of an interaction between two compounds [24,28].

The checkerboard assay evaluated at 24 h showed that the combination of carvacrol and cuminaldehyde decreased the SMIC_50_ values ~8-fold and 4-fold compared to the equivalent concentrations of cuminaldehyde and carvacrol when used alone, respectively (Supplementary Table S4). The MIC values of both terpenoids in combination were reduced to 0.06–0.25 mg/mL for carvacrol and 0.5–2.0 mg/mL for cuminaldehyde. The combination showed synergistic interactions (FICI values ranged 0.36 to 0.5) for 12 strains of *C. albicans* and indifferent interactions (FICI values between 0.62 and 1.0) for 4 *Candida* strains (A1, A2, A10, and A12).

### 3.4. Inhibition of Single- and Mixed-Species (C. albicans/bacteria) Biofilms by Plant Terpenoids

In order to evaluate the effect of terpenoids on microbial biofilm formation, biofilms formed by *C. albicans* (A5, A6, and ATCC10231) were treated with terpenoids as indicated in the Materials and Methods section. The formed biofilms were significantly reduced in the presence of carvacrol (Supplementary Figure S2) (*p* < 0.0001) and cuminaldehyde (Supplementary Figure S3), but at higher concentrations than that used in the case of yeast forms (Supplementary Tables S5 and S6). Carvacrol caused an 80% inhibition in biofilm formation at 2 mg/mL, while cuminaldehyde caused an 80% inhibition of biofilm formation at 6 mg/mL.

**Figure 3:**
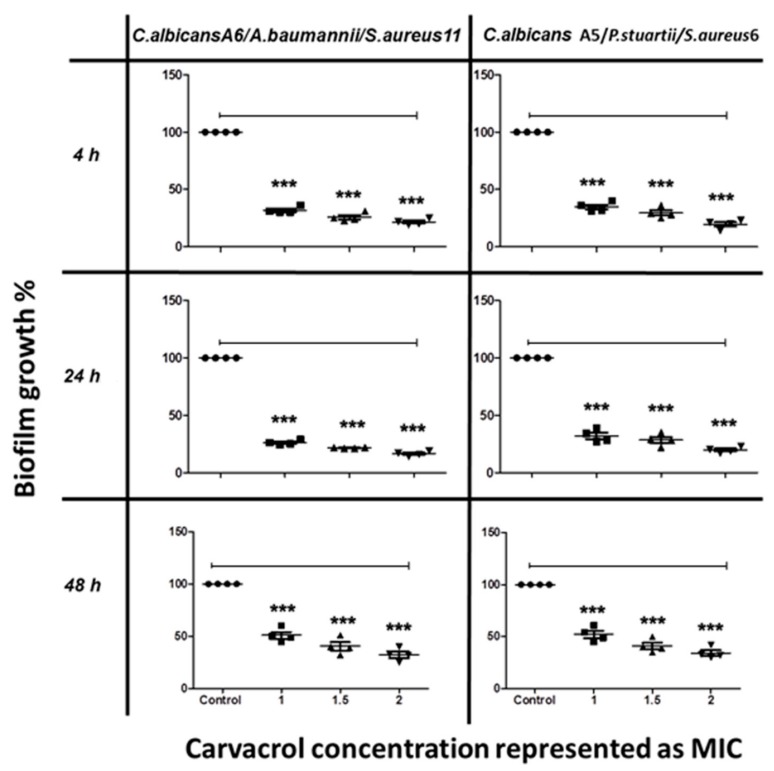
The inhibitory effect of carvacrol on *C. albicans*/bacteria in mixed biofilm formation at different time points, as determined by MTT viability assays. *C. albicans* cells were allowed to adhere onto 96 well microtiter plates at time points of 4 h, 24 h, and 48 h, followed by treatment with various concentrations of carvacrol at 1.0, 1.5, and 2.0 MIC prior to incubation at 37 °C. The anti-biofilm activity of the compound was assessed by measuring the metabolic activity of viable cells by MTT assay. The data were analyzed using one-way ANOVA and statistical significance was calculated with Dunn′s multiple comparison test. Significance level is indicated by asterisks. The data display the mean ± standard error (SEM) of four replicates.

The anti-biofilm activity of terpenoids was also tested on *C. albicans* mixed with co-isolated bacteria. Mixed-species biofilm formation was significantly inhibited by carvacrol in a concentration-dependent manner, with more than 75% inhibition at 2 mg/mL (Figure 3).

**Figure 4:**
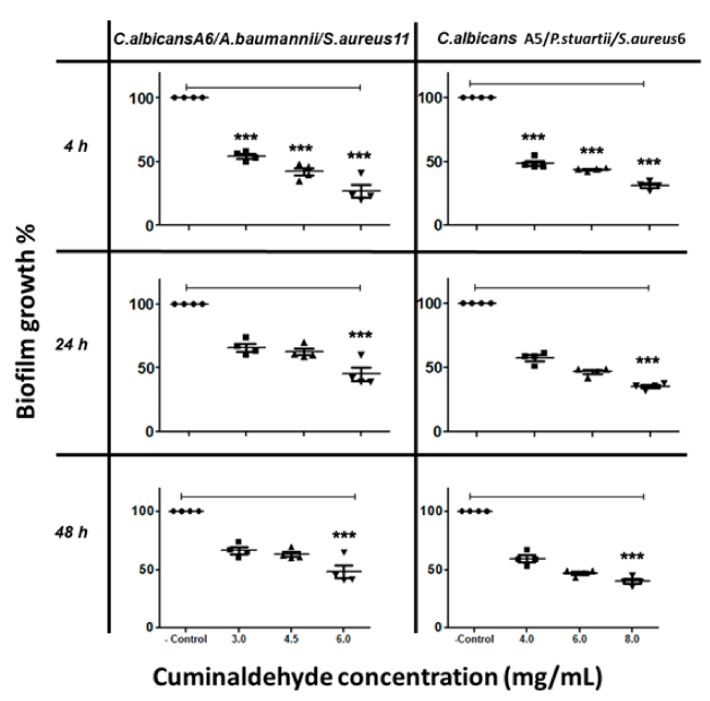
The inhibitory effect of cuminaldehyde on *C. albicans*/bacteria in mixed-species biofilm at different time points, as determined by MTT reduction assays. *C. albicans* cells were allowed to adhere onto 96 well plates at different times (4 h, 24 h, and 48 h), followed by treatment with various cuminaldehyde concentrations prior to incubation at 37 °C. The anti-biofilm activity of the compound was assessed in terms of metabolic activity by MTT assay. The data were analyzed using one-way ANOVA and statistical significance was calculated with Dunn′s multiple comparison test. Significance level is indicated by asterisks. The data display the mean ± standard error (SEM) of four replicates.

On the other hand, cuminaldehyde at 6 mg/mL caused a 60% inhibition to biofilm formed by *C. albicans* A6/*S. aureus*/*Acinetobacter baumannii* (Figure 4), and at 8 mg/mL caused 62% inhibition to biofilm formed by *C. albicans* A5/*P. stuartii*/*S. aureus* (Figure 4).

The results indicated that carvacrol interrupted the interactions among the mixed species and inhibited the biofilm formation at concentrations lower than that needed for cuminaldehyde. Carvacrol showed better anti-biofilm effect against single- and mixed-species biofilms during the first phases (adhesion phase 4 h and initial colonization 24 h) compared to the maturation phase (48 h).

### 3.5. Optimum Inhibition of C. albicans by Combination of Yeast and Plant Terpenoids

**Figure 5:**
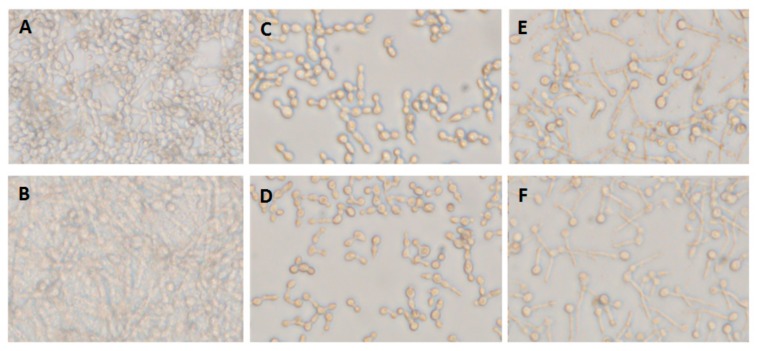
Dual inhibitory effect of farnesol and carvacrol on single- and mixed-species *C. albicans* biofilm formation. (**A**) Single-species *C. albicans,* A5 biofilm formed after 24 h. (**B**) Mixed-species biofilm formed by *C. albicans* A5, *P. stuartii* 3, and *S. aureus* 6 (A5/Ps3/Sa6). (**C**) Single-species biofilm treated with farensol at 0.7 mg/mL in combination with carvacrol at 0.25 mg/mL. (**D**) Single-species biofilm treated with farensol at 0.7 mg/mL in combination with carvacrol at 0.5 mg/mL. (**E**) Mixed-species biofilm treated with farensol at 0.7 mg/mL in combination with carvacrol at 0.25mg/mL. (**F**) Mixed-species biofilm treated with farensol at 0.7 mg/mL in combination with carvacrol at 0.5 mg/mL.

Carvacrol effectively inhibited both *Candida* forms in lower doses, and had the capability to penetrate the formed biofilms. Thus, a combination of farnesol, a hyphae-specific inhibitor, and carvacrol showed non-selective inhibition effect on *C. albicans* either in yeast (Table 2) or hyphae/biofilm forms (Figure 5). The combined treatment was effective against hyphae formed by *C. albicans* both incubated alone (Figure 5A,C,D) or when mixed with co-isolated bacteria (Figure 5B,E,F). The interaction was synergistic.

## 4. Discussion

The MIC values for amphotericin B found in this study were much higher than those reported in various studies for *Candida* species [29,30,31] which may be attributable to the capacity of *C. albicans* to develop genetic mutations [32,33], leading to biodiversity [34].

The selective inhibition by farnesol of *Candida* hyphae formation but not the yeast form was in accordance with several reported data [35,36,37]. Farnesol treatment may induce *Candida* apoptosis, disordered mitochondria due to the production of reactive oxygen species [11,38], change in cell development [39], and necrosis [40]. Although farnesol caused inhibition of biofilm formed by single *C. albicans* species, as previously reported [41,42], it did not reduce the biofilm formed by mixed species. The presence of bacteria is known to increase the resistance of such biofilms [22].

The higher susceptibility of *C. albicans* isolates to carvacrol was in accordance with the Raut et al. 2013 study [15] which reported that carvacrol exerts anti-*Candida* activity by interfering with endoplasmic reticulum integrity [43] and alteration in calcium homeostasis [44], ergosterol biosynthesis [45], and plasma membrane integrity [46]. Furthermore, our data showed that the combination of carvacrol and cuminaldehyde caused synergistic interactions (FICI values ranged from 0.36 to 0.5) to most tested *C. albicans* strains. The results obtained were in accordance with previous studies confirming the synergistic activity of essential oils, such as lavender oil in combination with *Melaleuca alternifolia* oil [47] and lavender oil in combination with *Pelargonium graveolens* oil [48].

Both terpenoids also showed strong inhibition of biofilm formation and penetration capability to formed biofilm. The anti-biofilm activity of terpenoids may be attributed to membrane damage, as well as inhibition of oxidative phosphorylation and respiratory chain functions [49]. Terpenoid-mediated changes in membrane permeability and fluidity can result in the degradation of cell walls, and hence affect the adherence of *C. albicans* to solid surfaces [50]. Furthermore, carvacrol itself at 2 mg/mL showed ~ 75% inhibition of mixed-species biofilm, which was consistent with previous studies of the anti-*Candida* activity of carvacrol [43,51,52].

Although some studies have shown that mature mixed biofilms of bacteria and *Candida* exhibit enhanced resistance compared to single-species biofilms [53], the terpenoid compounds used in this study, in particular carvacrol, caused strong inhibition to the biofilm formed by *C. albicans* A6/*S. aureus*/*A. baumannii*, despite the cohabitation of multidrug-resistant *A. baumannii*.

It is important to note that the majority of anti-fungal agents had limited anti-biofilm activity. It was reported that *Candida* biofilms were 30 to 2000 times more resistant to various antifungal agents compared to their planktonic counterparts [54,55]. The mechanisms by which biofilms develop antimicrobial resistance are complex and multifactorial. These include altered gene expression following surface attachment, reduced growth rates, variable nutrient availability that induces changes in phenotype, and the presence of extracellular polymeric substances that impedes the penetration of antimicrobial agents to biofilm [56].

Some studies have shown that combinations of plant essential oils or their terpenoid components with commercial antimycotics are known to result in synergistic antifungal activities [57,58]. Interestingly, similarly to our synergistic data using carvacrol and farnesol, carvacrol has been reported to have synergistic interactions with fluconazole against the planktonic and biofilm forms of *C. albicans* [59]. Furthermore, carvacrol has been classified as GRAS (generally recognized as safe) and approved for food use [60], as has cuminaldehyde [61]. The results from this study can be employed both for the prevention of biofilm formation by single or mixed species and for their treatment.

## 5. Conclusions

As concluding remarks, terpenoids showed effective inhibition of different *Candida* forms, including yeast, hyphae, and biofilm, based on the type of terpenoid used. Endogenous terpenoid (farnesol) produced by *C. albicans* yeast effectively inhibited *Candida* hyphae formation, but this was not the case when *C. albicans* was mixed with bacteria or existed as *Candida* hyphae/biofilm, indicative of a lack of biofilm-penetration capability. Furthermore, farnesol required ~12–50 times its hyphae inhibitory concentration to inhibit the yeast form. Exogenous plant terpenoids present in our food and condiments showed non-selective inhibition of *Candida* forms including yeast and hyphae. Combination of exogenous terpenoids with endogenous terpenoids showed a synergistic non-selective inhibition effect on both *C. albicans* forms. Furthermore, the combination showed promising reduction of *Candida* biofilm, indicative of a penetration capability when compared to available antifungal agents. Importantly, the concentration of the combined terpenoids was significantly reduced ~10–20 times, a non-toxic concentration available in regular food and condiments, as reported previously. Thus, the terpenoids from this study, in particular carvacrol in combination with farnesol, can be used as protective and therapeutic agents against *C. albicans* in its different forms. Moreover, the combined terpenoids can be used as a promising safe preservative for prosthetic devices.

## Figures and Tables

**Table 1:** *Candida* and bacterial strains employed in the study.

*Candida*		Bacteria	
Strain	Symbol	Strain	Symbol
*Candida albicans 1*	A1	*Escherichia coli*	Ec2
*Candida albicans 2*	A2	*Escherichia coli*	Ec4
*Candida albicans 3*	A3	*Escherichia coli*	Ec13
*Candida albicans 4*	A4	*Providencia stuartii*	Ps2
*Candida albicans 5*	A5	*Providencia stuartii*	Ps3
*Candida albicans 6*	A6	*Acinetobacter baumannii*	Ab7
*Candida albicans 7*	A7	*Acinetobacter baumannii*	Ab11
*Candida albicans 8*	A8	*Proteus mirabilis*	Pm16
*Candida albicans 9*	A9	*Proteus mirabilis*	Pm18
*Candida albicans 10*	A10	*Proteus mirabilis*	Pm19
*Candida albicans 11*	A11	*Proteus mirabilis*	Pm21
*Candida albicans 12*	A12	*Staphylococcus aureus*	Sa6
*Candida albicans 13*	A13	*Staphylococcus aureus*	Sa11
*Candida albicans 14*	A14	*Staphylococcus aureus*	Sa24
*Candida albicans 15*	A15	*Staphylococcus epidermidis*	Se3
*Candida albicans*	ATCC10231		

**Table 2:** Interactions (indicated by FIC indices) of carvacrol in combination with farnesol against *C. albicans* after 48 h of incubation.

Strains	MIC (mg/mL)			FICI*	Interactions
	Carvacrol	Farnesol	Carvacrol/Farnesol		
**ATCC10231**	0.5	150	0.03 + 0.2	0.06	Synergy
**A1**	0.5	16.8	0.125 + 1.2	0.33	Synergy
**A2**	0.5	33.6	0.03 + 1.2	0.10	Synergy
**A3**	1	33.6	0.06 + 0.2	0.06	Synergy
**A4**	0.25	33.6	0.03 + 0.4	0.13	Synergy
**A5**	1	33.6	0.125 + 1.2	0.17	Synergy
**A6**	0.5	33.6	0.06 + 1.2	0.16	Synergy
**A7**	0.5	33.6	0.125 + 0.4	0.26	Synergy
**A8**	1	16.8	0.25 + 0.4	0.28	Synergy
**A9**	1	33.6	0.25 + 0.4	0.26	Synergy
**A10**	0.5	33.6	0.06 + 0.8	0.15	Synergy
**A11**	1	33.6	0.25 + 0.8	0.28	Synergy
**A12**	0.5	33.6	0.125 + 1.2	0.29	Synergy
**A13**	1	33.6	0.25 + 0.8	0.28	Synergy
**A14**	0.5	16.8	0.125 + 1.2	0.33	Synergy
**A15**	1	33.6	0.125 + 1.2	0.15	Synergy

## Supplementary Materials

Tables S1–S6 and Figures S1–S3 are available as Supplementary data at https://www.mdpi.com/2218-273X/10/2/342/s1.
