# Erratum: Touil, H.F.Z. et al. Optimum Inhibition of Amphotericin-B-Resistant *Candida albicans* Strain in Single- and Mixed-Species Biofilms by *Candida* and Non-*Candida* Terpenoids. *Biomolecules* 2020, *10*, 342

**DOI:** 10.3390/biom10060847

**Published:** 2020-06-02

**Authors:** Hidaya F.Z. Touil, Kebir Boucherit, Zahia Boucherit-Otmani, Ghalia Khoder, Mohamed Madkour, Sameh S. M. Soliman

**Affiliations:** 1 Laboratory Antibiotics Antifungals: Physico-Chemical, Synthesis and Biological Activity (LapSab), Tlemcen University, Tlemcen B.P 119, Algeria; hidayet.touil@gmail.com (H.F.Z.T.); boucheritkebir@yahoo.fr (K.B.); z_boucherit@yahoo.fr (Z.B.-O.); 2 Sharjah Institute for Medical Research, University of Sharjah, Sharjah PO. Box 27272, UAE; gkhoder@sharjah.ac.ae (G.K.); mmadkour@sharjah.ac.ae (M.M.); 3 Department of Pharmaceutics and Pharmaceutical Technology, College of Pharmacy, University of Sharjah, Sharjah PO. Box 27272, UAE; 4 Department of Medical Laboratory Sciences, Collage of Health Sciences, University of Sharjah, Sharjah PO. Box 27272, UAE; 5 Department of Medicinal Chemistry, College of Pharmacy, University of Sharjah, Sharjah PO. Box 27272, UAE; 6 Department of Pharmacognosy, College of Pharmacy, University of Zagazig, Zagazig 44519, Egypt

The authors wish to make the following correction to this paper [1].

The last name of Dr. Ghalia Khoder was incorrect in the published paper in *Biomolecules* [1].

Therefore, this information was changed by the Biomolecules Editorial Office from:

Hidaya F.Z. Touil ^1^, Kebir Boucherit ^1,2^, Zahia Boucherit-Otmani ^1^, Ghalia Kohder ^3,4^, Mohamed Madkour ^3,5^ and Sameh S. M. Soliman ^3,4,6,^*

To:

Hidaya F.Z. Touil ^1^, Kebir Boucherit ^1,2^, Zahia Boucherit-Otmani ^1^, Ghalia Khoder ^3,4^, Mohamed Madkour ^3,5^ and Sameh S. M. Soliman ^3,4,6,^*

We apologize for any inconvenience caused to the readers. The manuscript will be updated, and the original will remain online on the article webpage, with a reference to this Erratum.

## References

[1] Touil H.F.Z., Boucherit K., Boucherit-Otmani Z., Kohder G., Madkour M., Soliman S.S.M. (2020). Optimum Inhibition of Amphotericin-B-Resistant *Candida albicans* Strain in Single- and Mixed-Species Biofilms by *Candida* and Non-*Candida* Terpenoids. Biomolecules.

