# Supplementary material for: Optimum Inhibition of Amphotericin-B-Resistant Candida albicans Strain in Single- and Mixed-Species Biofilms by Candida and Non-Candida Terpenoids
Source: Biomolecules. 2020 Feb 21;10(2):342. doi: 10.3390/biom10020342 (PMC7072433; doi:10.3390/biom10020342)
Supplement: Supplementary file 1 [file biomolecules-10-00342-s001.pdf]

Supplemetary Tables

Supplementary Table 1. Minimum inhibitory concentrations (MIC) and minimum fungicidal concentrations (MFC) of farnesol against *C. albicans* clinical isolates and TCC10231 strains.

**Strains**	**MIC (mg/mL)**	**MFC (mg/mL)**
A1	16.8	33.6
A2	33.6	67.2
A3	33.6	33.6
A4	33.6	67.2
A5	33.6	67.2
A6	33.6	33.6
A7	33.6	67.2
A8	16.8	33.6
A9	33.6	67.2
A10	33.6	33.6
A11	33.6	33.6
A12	33.6	33.6
A14	16.8	16.8
A13	33.6	33.6
A15	33.6	33.6
ATCC10231	33.6	33.6

Supplementary Table 2. Minimum Inhibitory Concentrations (MIC) and Minimum Fungicidal Concentration (MFC) of carvacrol and cuminaldehyde against *C. albicans* ATCC10231 and *C. albicans* strains isolated from medical devices at the Tlemcen University Hospital.

***Candida* strains**	**MIC and MFC (mg/mL)**			
	**Carvacrol**		**Cuminaldehyde**	
	**MIC**	**MFC**	**MIC**	**MFC**
ATCC10231	0.5	0.5	4	4
A1	0.5	1	4	4
A2	0.5	0.5	4	4
A3	1	2	4	4
A4	0.25	0.5	2	3
A5	1	1	4	>4
A6	0.5	0.5	3	4
A7	0.5	0.5	4	4
A8	1	1	4	>4
A9	1	1	4	4
A10	0.5	0.5	4	4
A11	1	2	4	4
A12	0.5	0.5	2	2
A13	1	2	4	>4
A14	0.5	0.5	4	4
A15	1	2	4	4

Supplementary Table 3. Minimum Inhibitory Concentrations (MIC) and Minimum Bactericidal Concentrations (MBC) of carvacrol and cuminaldehyde against bacteria co-isolated with *C. albicans*.

**Bacteria strains**	**MIC and MBC (mg/mL)**			
	**Carvacrol**		**Cuminaldehyde**	
	**MIC**	**MBC**	**MIC**	**MBC**
Ec2	1	1	1	1
Ec4	1	1	1	1
Ec13	1	1	1	1
Ps2	1	1	1	1
Ps3	1	1	1	1
Ab7	1	1	1	1
Ab11	1	1	2	2
Kp18	1	1	1	1
Se2	1	1	3	3
Se3	1	1	1	1
Se9	1	1	2	2
Se10	1	1	>4	>4
Sa6	1	1	2	2
Sa11	1	1	2	2
Sa24	1	1	2	2
Pm16	1	1	2	2

Supplementary Table 4. Interactions of carvacrol and cuminaldehyde against mono-species biofilm formed by *C. albicans* clinical isolates ($SMIC_{50}$)

***Candida* strains**	**SMIC (mg/mL)**			
	**Carvacrol**	**Cuminaldehyde**	**Carvacrol / Cuminaldehyde**	**FICI**
**ATCC10231**	0.75	6	0.125/2	0.49
**A5**	1.5	7	0.25/1	0.30
**A6**	1.5	6	0.25/1	0.33
**A7**	1.5	6	0.125/1	0.24
**A2**	1.5	7	0.25/2	0.44
**A10**	1	7	0.06/2	0.34

$SMIC_{50}$: Sessile MIC, defined as the concentration that causes 50% reduction in optical density of the biofilms compared to the optical density of the untreated biofilm formed by the same isolates; FICI : fractional inhibitory concentration index

Supplementary Table 5. Interactions of carvacrol and cuminaldehyde against mono-species biofilm formed by *C. albicans* clinical isolates ($SMIC_{80}$)

***Candida* strains**	**SMIC (mg/mL)**			
	**Carvacrol**	**Cuminaldehyde**	**Carvacrol / Cuminaldehyde**	**FICI**
**ATCC10231**	1	6	0.25/1	0.41
**A5**	2	8	0.5/2	0.5
**A6**	2	8	0.25/1	0.25
**A7**	2	8	0.25/2	0.37
**A2**	2	8	0.5/4	0.75
**A10**	2	8	0.12/4	0.56

$SMIC_{80}$: sessile MIC, defined as the concentration that causes 80% reduction in biofilms formation.

Supplemtary Table 6. Interactions of carvacrol with farnesol against planktonic cells of *C. albicans after* 48h of incubation.

Strains	MIC (mg/mL)			FICI	Interactions
	Carvacrol	Farnesol	Carvacrol/ Farnesol		
**ATCC10231**	0.5	33.6	0.03+0.2	0.06	Synergy
**A1**	0.5	16.8	0.125+1.2	0.33	Synergy
**A2**	0.5	33.6	0.03+1.2	0.10	Synergy
**A3**	1	33.6	0.06+0.2	0.06	Synergy
**A4**	0.25	33.6	0.03+0.4	0.13	Synergy
**A5**	1	33.6	0.125+1.2	0.17	Synergy
**A6**	0.5	33.6	0.06+1.2	0.16	Synergy
**A7**	0.5	33.6	0.125+0.4	0.26	Synergy
**A8**	1	16.8	0.25+0.4	0.28	Synergy
**A9**	1	33.6	0.25+0.4	0.26	Synergy
**A10**	0.5	33.6	0.06+0.8	0.15	Synergy
**A11**	1	33.6	0.25+0.8	0.28	Synergy
**A12**	0.5	33.6	0.125+1.2	0.29	Synergy
**A13**	1	33.6	0.25+0.8	0.28	Synergy
**A14**	0.5	16.8	0.125+1.2	0.33	Synergy
**A15**	1	33.6	0.125+0.8	0.15	Synergy

Supplemetary Figures

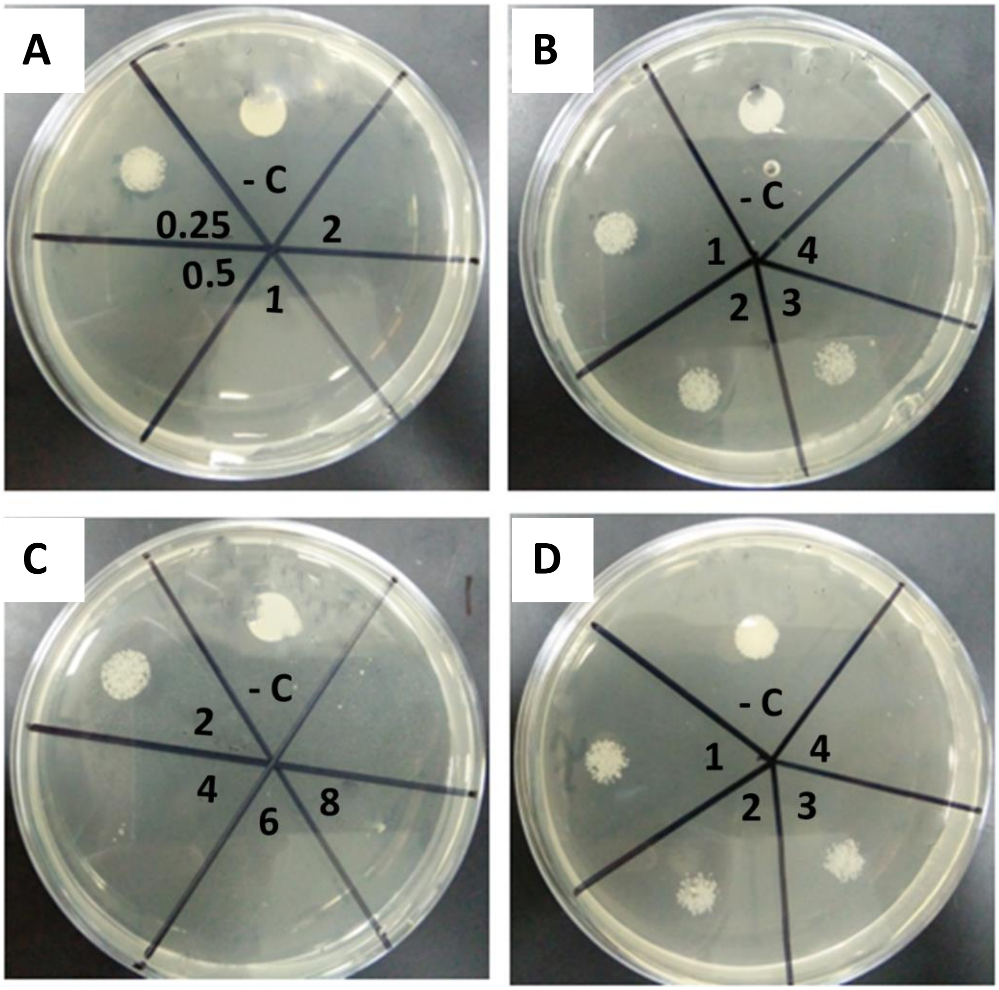

Supplementary Figure 1. Spot test assay of carvacrol and cuminaldehyde at different concentration on *Candida spp*. (A) Effect of carvacrol on *C. albicans* ATCC10231 reference strain. (**B**) Effect of carvacrol on *C. albicans* A5 clinical isolate. (**C**) Effect of cuminaldehyde on *C. albicans* ATCC10231. (**D**) Effect of cuminaldehyde on A5.

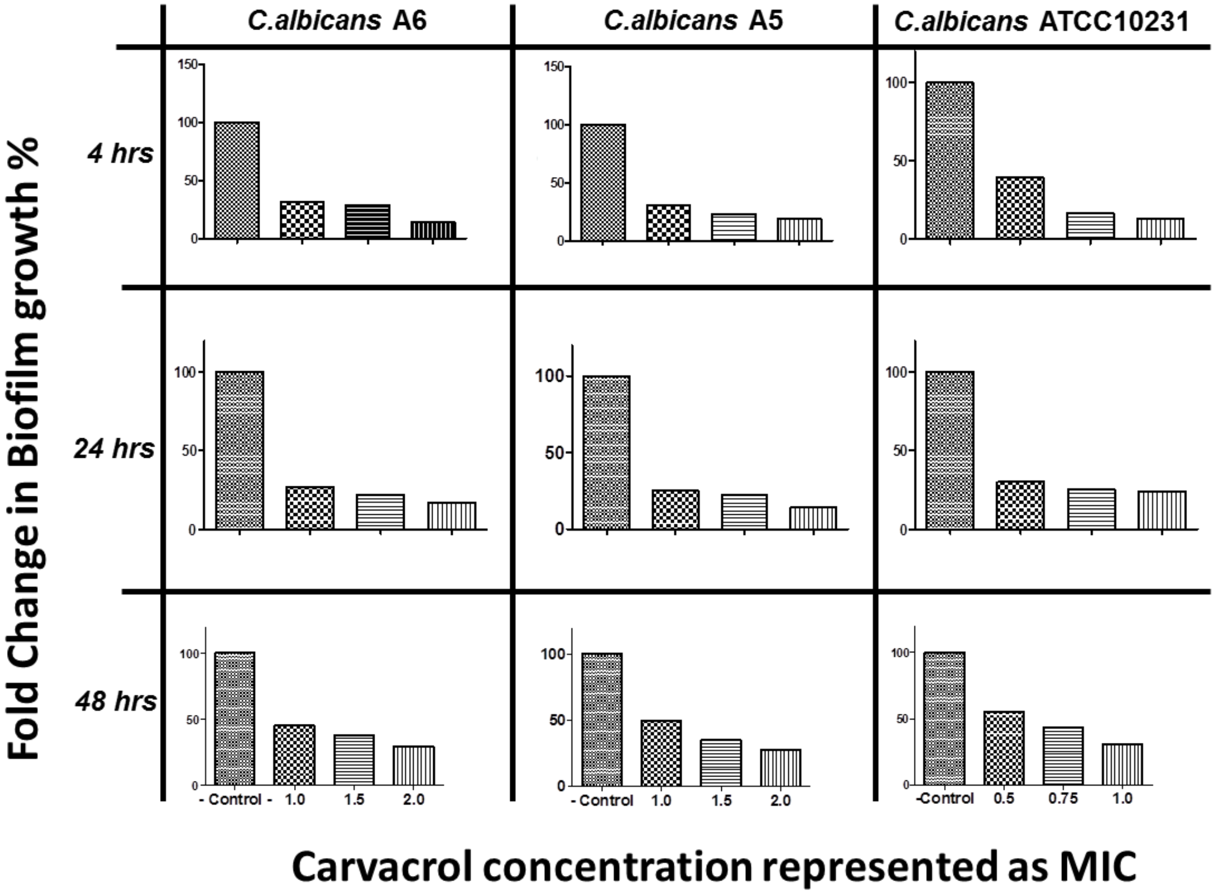

Supplementary Figure 2. The inhibitory effect of carvacrol on *C. albicans* biofilm formation at different time points and determined by MTT reduction assays. *C. albicans* cells were allowed to adhere to 96-well plates at different times (4h, 24h and 48h), followed by treatment with various MIC concentrations of carvacrol including 1.0, 1.5 and 2.0MIC prior to incubation at 37ºC. The antibiofilm activity of the compound was assessed in terms of metabolic activity by the MTT assay.

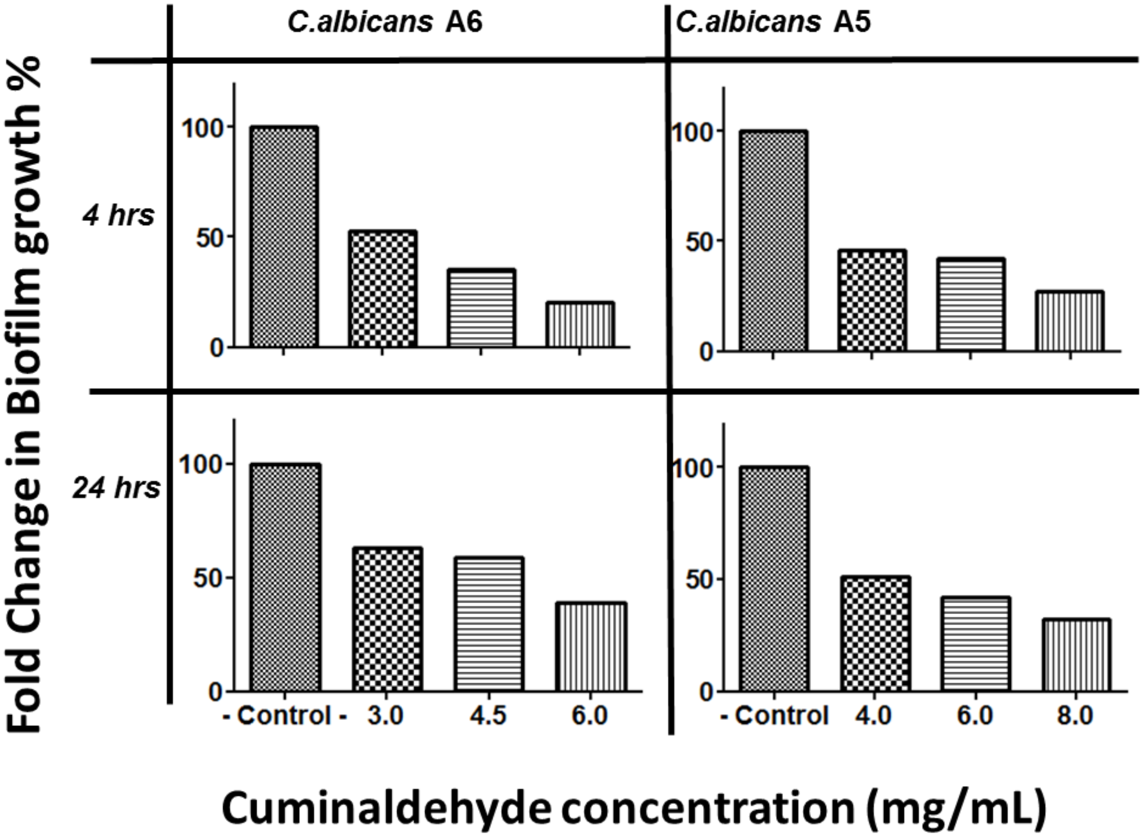

Supplementary Figure 3. The inhibitory effects of cuminaldehyde on *C. albicans* biofilm formation.
